# Furnaces

**Published:** 1876-10

**Authors:** 


### *FURNACES.*

How to warm our houses is a problem which seems no nearer to a satisfactory solution than it was twenty-five years ago. Every year some new furnace is put in the market, which, it is claimed, possesses all the requisites. It receives a thorough trial, and is in its turn cast aside to make room for the next "invention." And thus large sums are expended, in fact, wasted, in the effort to make ourselves comfortable in severe winter weather. Beside being the most wasteful and cheerless of all contrivances for warming houses, the furnace requires almost constant attention. It must be frequently replenished with coal; the grate must be often shaken; if the draught is opened the heat soon becomes too strong; if long closed it gives out but little heat; if the wind is in the wrong direction, but little outside air comes to the furnace, and the hot air does not ascend sufficiently to warm the rooms; if the water evaporates from the pan, the air seems dry, and there is a smell of gas. If there is water in the pan, the air in the rooms above becomes saturated with moisture, and the occupants are, perhaps, unconsciously, taking a vapor bath. When they leave the rooms and go out into the open air colds are almost inevitable, and this is the origin of very many of those diseases so terribly fatal in winter; as congestion of the lungs or brain, pneumonia, inflammatory rheumatism, and diphtheria. Not one person in fifty but thinks it is necessary to have a vessel of water on the stove or furnace to moisten the air. This is often a fatal mistake. The air is already tempered for our use, and we might as well try to improve the sun's rays. The atmosphere does not get dried out or burned in contact with the heated stove. A degree of heat that would affect it to that extent would be unendurable. It is difficult to convince people of the error of opinions they have always entertained, and never heard disputed. But whoever will take the trouble to investigate the matter, will find that there are more cases of illness during the winter among persons occupying rooms where the atmosphere is thus moistened, than among the same number of persons who breathe the natural air.

When the cold is not severe, the open grate fireplace is, no doubt, on every account, the best method of warming a house. In severe weather it is of little use. We should have to be lashed to a spit and revolved before the fire to keep from freezing. Of course we do not include the ventilating "Fire on the Hearth" stove in this remark, as that is something new to us.

Of all devices for warming rooms there is nothing better than the old-fashioned, sheet iron, brick-lined cylinder stoves. The base-burning, self-feeding stove now so much used, is not to be compared with it in economy, efficiency, safety or durability. Some of these stoves are constructed so that they will heat a lower and an upper room. Such a stove is superior to any furnace we have yet seen.

